# Notes and News

**Published:** 1888-10-13

**Authors:** 


### Notes and News.

Collected and Communicated.

According to Sir Henry Roscoe, German pharmacists are much superior to their English rivals in the skill and cunning they display in the discovery and preparation of new remedies.

The building of a new workhouse infirmary at Coventry will probably need an expenditure of £5,000, the annual payment for sinking fund and interest of which will be equal to a halfpenny rate.

A hospital for infectious diseases has been opened at Walker, three miles from Newcastle. It is built on the single storey pavilion system, and will accommodate 105 patient. The old building in Bath Lane will now be pulled down.

A copy of the second edition of Sir Francis Jervoise's pamphlet on "Infection," with Miss Nightingale's remarks, has been forwarded to us. Those who do not believe in the germ theory of disease may find it curious, and perhaps interesting, reading. Those who do believe will probably not read more than the first page.

Of 2,053 deaths registered in the sanitary district of Birkenhead during 1887 no fewer than 45 were returned as "uncertified." Notwithstanding this the cause of death was in most cases either known or guessed at, inasmuch as all of them were registered under one or other of the usual denominations suggested by the Registrar-General.

According to a writer in the *Field*, English bloodhounds are now of very little use in tracking criminals. From want of use their power of discriminating scent is said to be very much diminished, though not entirely lost. This is quite in accordance with scientific experience. No doubt a course of training would develop in bloodhounds the sense which for want of such training is more or less in abeyance.

The inhabitants of Forres have had £5,000 offered them towards building a cottage hospital, on condition that they will bestir themselves and provide what else is necessary. At a meeting held lately in the Court House, Provost Burn presiding, a subscription list amounting to £1,171 14*s*. was shown, and it was announced that the Town Council had granted a favourable site. Surely Forres will soon have its hospital.

Mr. John Fleming is to be congratulated upon the completion of his philanthropic work in memory of his wife. The hospital which he has erected on the Moor Edge, Newcastle, is a model building which combines use and ornament, and it will prove as great an advantage to the poor as it is an adornment to the city. It is arranged on an admirable system, and is replete with every modern appliance. Lord Armstrong performed the opening ceremony on September 26th, so that the children who have hitherto been cooped up in the city may now enjoy the breezes of the moor and every care that wealth can give them.

On September 29th, the corner-stone of the Hartlepool Hospital extension was laid by the Rev. J. Burdon. The hospital was inaugurated in 1864 by the late Rowland Burdon, and is no longer large enough for the town; the extension will provide for just double the present number of beds, say fifty in all. Mr. Cardwell amidst much applause placed a cheque for £10 on the stone, as a special contribution from the workmen of the Central Marine Engineering Company.

The hospital smack *Queen Victoria*, has been successfully launched, and will soon be despatched to the North Sea fishing-grounds. The vessel has been constructed with a view to the special service on which she is to be engaged, and has on board all necessary appliances for the reception of any bad case requiring immediate surgical assistance. She is in charge of skilled medical attendants, and will prove a great blessing to the hardy fellows who man our trawling fleets.

The munificence of the Whitworth Legatees is a copious stream. At a meeting held at Owens College on October 2nd, they proposed to give to the city of Manchester a general hospital, which they offer to build and partially endow, and which shall be under the control of the college, and in direct communication with the medical school. The cost of the hospital is estimated at £35,000, and £1,000 a-year is to be the endowment.

The "Mutual Friendly Aid Society" has a good name and does good work. It is mutual, it is friendly, it is for purposes of aid. What better title could be desired? Last year it raised nearly £400 for distribution among several medical charities at the East End of London. In return it received tickets of admission which we may be sure it distributed with full knowledge of the necessities of the cases sent for hospital treatment. The *modus operandi* of the Society is one which might well be followed by working men throughout the metropolis and the country. It consists in the raising of a "fund" by means of subscriptions, donations, and otherwise, for the purchase of life governorships at certain hospitals. Those governorships, it is known, carry several privileges, among the rest a definite number of annual hospital tickets. The life governors are taken from among the working men members of the Society, and a certain amount of advisory supervision can be exercised over them by their fellow-members. The idea is excellent, and, as we have said, is worthy of extensive adoption.

The annual sermons on behalf of the Surrey County Hospital were delivered by the Rev. W. S. Sanders, at St. Nicolas Church, on Sunday last. Taking for his text in the morning Acts x. 38, "How God anointed Jesus of Nazareth with the Holy Ghost and with power: who went about doing good, and healing all that were oppressed of the devil; for God was with him," the rev. gentleman made an earnest appeal on behalf of the hospital funds, and remarked that he thought the following figures would speak for themselves: During the year 1887, 4,608 persons found relief from their sufferings, either within the walls of the hospital or from services rendered by those who were connected with the institution. Of that number no less than 114 persons in Guildford alone were in-patients, many of whom were sent forth healed and cured of their diseases, and 940 were Guildford out-patients. How could they find words expressive of thanks to God for so great a work as this? Let them exercise their sympathies and kindness by giving freely to so excellent an institution, and thus by striving to heal the sufferings of their brethren, come like good Samaritans to the rescue of the oppressed. The services were all largely attended. The offertories amounted to £31 16*s*. 7*d*.—a slight falling off as compared with last year—but this is no doubt accounted for by the fact that a large number of parishioners are at this season away from Guildford.

THE following vacancies are announced this week, the amount of salary in each case being given after the name of the institution :—

**Resident Medical Officers.**

North-West London Hospital. Senior Medical Officer.
Western Dispensary, Rochester Row. Medical Officer.—£100.
St. Marylebone General Dispensary. Medical Officer.—£105.
Paddington Green Children's Hospital. House Surgeon.—£50, with board, etc.
Liverpool Infirmary for Children. Assistant House Surgeon.—Board, etc.
Rubery Hill Asylum. Assistant Medical Officer.—£100, with board, etc.
Mile End Infirmary. Assistant Medical Officer.—£100, with board, etc.
Royal Albert Asylum, Lancaster. Assistant Medical Officer.—£120, with board, etc.
Hospital for Consumption and Diseases of the Chest. House Physician.-Board, etc.
Weston-super-Mare Hospital. House Surgeon.—£60.

**Non-resident Medical Officers.**

Cookham Union. Medical Officer.—£115.

---

THE informal gathering at the residence of Dr. Barron, Old Elvet, Durham, on September 22nd, should lead to great things. A number of members of friendly societies met to consider the suggestion that a Friendly Societies' Convalescent Home for Northumberland and Durham should be set up, and a resolution cordially approving of the project was adopted. A committee was appointed, with Mr. J. J. Robinson as hon. secretary, and another meeting will be held on October 20th. It must be explained that Dr. William Robinson, of Stanhope, was the first to move in the matter. One essential part of the scheme is that the Home should be managed by the friendly societies themselves. There certainly appears to be room for such an institution. The cost to individual lodges should not be heavy, and members of friendly societies would maintain their admirably-independent position by going to recruit health in their own Home. Some of the correspondents of the *Durham Chronicle* think it would be better to built a home open to all the working classes, not merely to member of friendly societies.

---

THE Marquis of Bute, accompanied by the Marchioness of Bute and the Earl of Dumfries, opened the hospital at Merthyr last week, Mr. C. H. James, High Constable, having the conduct of the proceedings at the building. The processional display was very effective. The Lord Bishop of Llandaff offered prayer, and a silver key was presented to his lordship, who addressed the crowd from the steps, and declared the institution open. The ground was kept by local detachments of the rifle volunteers, commanded by Lieutenant-Colonel Lewis. Among the speakers were Mr. W. L. Daniel, Mr. Thomas Williams, Mr. Edward Martin (Dowlais), Mr. W. Evans (Cyfarthfa), and Mr. Frank James, who announced that Lord Windsor would subscribe £200, and that the licensed victuallers had sent a first donation of £25. The hospital is prettily situated, and stands back a little distance from the road. It is built with the best Ebbw Vale hand-pressed bricks and dressings of puff terra-cotta, and to prevent dampness White's hygienic cement is used in all outside walls. On the ground floor there are two wards 44 ft. by 26 ft., and a special ward 26 ft. by 18 ft. Each ward has attached to it the necessary lavatories, nurses' sitting-rooms, and sculleries, with inspection windows to each. In addition to the wards there are also on this floor an operation-room, splint-room, instrument-room, and committee-room. The area of the site being somewhat limited, advantage has been taken of the great fall of the ground to form a basement, with a large kitchen, 28 ft. by 18 ft. ; scullery, wash-house, foul linen-room, with shoot from nurses' scullery ; also large larders, coal cellar, and ice-house. This hospital should supply a great want, as accidents amongst the workmen of Merthyr and Dowlais are very common.

OUR neighbours in Essex have been having what might be called a warm discussion about the site of a proposed infectious hospital. This is a subject which is frequently warmly discussed, but the site was objected to not only because you can stand in the middle of it and throw stones into three pathways, but because it is damp, and liable to be flooded, and close to some noisy mills. However, seven of the Sanitary Committee approved of the site, and outvoted the five malcontents.

---

THE extensive structural alterations which have been going on at the Northern Infirmary, Inverness, are now finished, and the patients are back in the large light wards caused by the improvements. The surplus from the Northern Meeting Games will furnish one ward, but funds are urgently needed. Considering that this is the infirmary for the whole of the North of Scotland, it should not need to beg. Where is Mr. Carnegie, who constantly visits this neighbourhood, and who has determined not to die rich ?

---

THE new dispensary in connexion with the Glasgow Sick Children's Hospital has been formally opened by the Duke of Montrose. The dispensary, which has been built from plans prepared by Mr. James Sellars, architect, consists of a waiting-hall, having on one side the physicians' and on the other the surgeons' rooms, all of which are most commodious. By means of a sum of £4,000 set apart from the funds realised at the fancy fair four years ago, and certain special legacies and donations, the directors were happily able to open the dispensary entirely free of debt. No subscriber's lines will be necessary for those attending the dispensary, but the institution is in strict and constant communication with the Charity Organisation, which will prevent abuse of its privileges.

---

A SPECIAL meeting for prayer to inaugurate the present session of the Zenana Mission was held on Wednesday, 3rd inst., at eleven a.m., when the Rev. Julius Griffith (brother of the founder and hon. secretary, Dr. G. de G. Griffith) presided. After singing and prayer, the Rev. J. G Rainsford, father of one of the students who last year passed her examinations with honours, and is to leave for Srinagar on the 10th inst., under the auspices of the C.E.Z.M.S., read a few verses from Col. iii., and delivered an impressive address to the students on the importance of the abundant in-dwelling of the Word of God. After singing, the Rev. J. Griffith closed with prayer, in which Miss Rainsford (who was present) and Miss Parslee, a former student, who is returning to her work in Jandiala, were specially and affectionately remembered.

---

THE merriment of the daily press is very lively over the undignified appearance made by certain medical practitioners of Birmingham. It appears that the "Medical Institute" at that redoubted centre of mixed politics and universal wares has actually been the scene of a meeting of homœopathic practitioners. How the homœopaths obtained the use of the rooms it is not quite clear, although it is hinted that there was a want of frankness somewhere. They did, however, obtain permission to hold their meeting by some means, and immediately thereafter the whole world is informed of the fact. The result has been a considerable explosion in orthodox medical circles. Four members of the Institute, it is reported, at once handed in their resignations. How many more may still resign it is impossible to say. A daily contemporary suggests the desirability of thoroughly fumigating the Institute before it is again used for orthodox purposes. We cannot but think the Birmingham medical men have shown a want of sense and dignity in their precipitate excitement. If a mistake has been committed, all that is required is to make sure of its non-repetition.

